# Human Mpox (formerly monkeypox): The New Great Imitator?

**DOI:** 10.4269/ajtmh.22-0509

**Published:** 2022-12-12

**Authors:** Samantha Pérez-Cavazos, Edgar Pérez Barragán

**Affiliations:** ^1^ Department of Hospital Epidemiology and Infection Prevention, Hospital Christus-Muguerza Betania, Puebla, Puebla, Mexico;; ^2^ Infectious Diseases Department, Hospital de Infectología, Centro Médico Nacional La Raza, Mexico City, Mexico

Since early May 2022, cases of mpox (formerly monkeypox) have been reported in the United Kingdom and subsequently in countries where the disease is not endemic.^1^^,^^2^ Classic features of the disease include macules, papules, umbilicated vesicles with a necrotic center, as well as pustular and crusting lesions. Nevertheless, new clinical presentations of mpox infection have been identified.^3^

**Figure 1.**
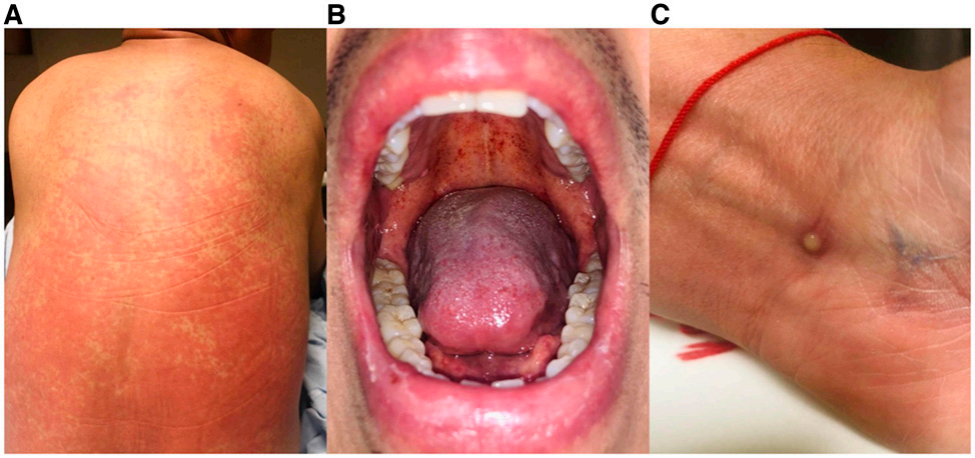
(**A**) Confluent pinkish maculopapular rash predominantly on the back. (**B**) Stippled petechial hemorrhages in the soft palate (Forchheimer’s spots). (**C**) Vesiculopustular lesion on the left wrist, compatible with classic mpox dermatosis.

We report the case of a 38-year-old male living with HIV who presented with a clinical course of 4 days of evolution with asthenia, adynamia, fever, and rash. On physical examination, generalized and confluent maculopapular rash predominantly on the back (Figure 1A), cervical and inguinal lymphadenopa thies, and Forchheimer’s spots were observed (Figure 1B). Subsequently, classic mpox pustules appeared (Figure 1C). Polymerase chain reaction (PCR) for measles and rubella were negative, and PCR for mpox was positive.

The present mpox outbreak has been the largest outbreak in the history of the disease. The situation has been further complicated due to its unusual clinical presentation and evolution, including unusual morphologies and lesion sites.^3^^,^^4^ Although confluent maculopapular rash and Forchheimer’s spots have been reported as classic signs of rubella or measles infections, this is the first report of mpox with this clinical presentation. We may now have a new “great imitator” disease.
